# Attachment and Dyadic Forgiveness in Individuals in Same-Sex Couple Relationships

**DOI:** 10.3390/ijerph191811152

**Published:** 2022-09-06

**Authors:** Mónica Guzmán-González, Nikolás Bekios, Josefa Burgos, Camila Obregón, Benjamín Tabilo, Giulia Casu

**Affiliations:** 1 School of Psychology, Universidad Católica del Norte, Avenida Angamos 0610, Antofagasta 1240000, Chile; 2 Department of Psychology, University of Bologna, 40127 Bologna, Italy

**Keywords:** attachment anxiety, attachment avoidance, dyadic forgiveness, LGB individuals, same-sex relationships

## Abstract

(1) Background: There is abundant evidence linking attachment insecurity to lower levels of interpersonal forgiveness. However, this association has been almost exclusively explored in people in a different-sex couple relationship, and there is little evidence referring to gay, lesbian, and bisexual (LGB) people in a same-sex relationship. The present study examines the association between romantic attachment and dyadic forgiveness in individuals involved in a same-sex couple relationship. (2) Methods: A total of 248 participants (52.8% female) aged 18–67 years (mean age: 31.02 ± 9.39) completed the Experiences in Close Relationships questionnaire and the Transgression-Related Interpersonal Motivation Inventory. (3) Results: Structural equation modeling showed that attachment avoidance was more strongly associated with dyadic forgiveness than attachment anxiety. Higher levels of attachment anxiety and avoidance were both linked to higher levels of avoidance motivation in response to partner transgressions, and higher attachment avoidance was linked to lower benevolence motivation. Multigroup analyses indicated a gender-specific association between attachment avoidance and revenge motivations, which was positive and significant among men only. (4) Conclusions: In the present study, we have identified that attachment avoidance plays a more prominent role in the tendency to forgive in same-sex couples. Implications at both theoretical and clinical practice levels are discussed.

## 1. Introduction

One of the conceptual formulations that allow us to understand couple functioning is Bowlby’s attachment theory [1], which refers to the need to establish close bonds with others as a way of obtaining a sense of security. One aspect that has been studied from this conceptual framework is the ability to cope with relational transgressions (defined as behaviors that violate relational rules and elicit negative emotional responses in the victim [2,3]) through forgiveness [4,5]. Forgiveness can be conceptualized as a process that involves the gradual decrease in negative reactions toward an offending other and the gradual development of more positive emotions, thoughts, and behaviors toward that person [6]. When referring to the tendency to forgive inside a specific relationship, this is called dyadic forgiveness [7].

There is abundant evidence linking attachment insecurity to lower levels of interpersonal forgiveness [4]. However, this association has been almost exclusively explored in people in a different-sex couple relationship, and there is little evidence referring specifically to gay, lesbian, and bisexual (LGB) people in a same-sex relationship [8,9]. The paucity of evidence linking attachment characteristics to forgiveness in the LGB population could be based on the assumption that the differences in functioning and dynamics between same- and different-sex couple relationships are fewer than similarities [8]. However, the LGB population has been historically and continues to be subject to prejudice and discrimination [10], which may bring specific stressors and challenges that can spill over into the quality of one’s relationship [11]. These LGB-specific experiences of prejudice and discrimination could then have an impact on the quality of couple relationships either negatively, diminishing the relational well-being [12] or positively, as sources of resilience that help same-sex couples cope with minority stress [13]. Despite this, the identification of peculiarities of same-sex couple relationships needs further investigation. Hence, the present study aims to contribute to this gap by investigating the association between attachment insecurity and dyadic forgiveness in individuals involved in same-sex couple relationships. Secondarily, the current research explores whether this association is invariant across gender. While gender differences in levels of forgiveness have been extensively investigated, although with inconclusive results [14,15], the moderating role of gender in the attachment–forgiveness link has been rarely investigated and limited to heterosexual individuals and couples [16,17,18].

### 1.1. Attachment and Forgiveness

Attachment theory states that schemes about oneself and what can be expected of others are formed from the quality of repeated experiences with significant others [1]. These experiences, called internal working models, are internalized as representations of the self and the world that guide the way one functions in various contexts, especially those that promote intimacy [1]. The model of self refers to beliefs about one’s own ability to mobilize support and feel loved by others. The model of others includes representations of the capacity of others to respond to one’s own demands [19]. These models are related to the levels of attachment anxiety and avoidance, respectively, and account for individual differences in attachment security.

Attachment anxiety refers to the degree of concern about abandonment from attachment figures due to a negative model of self [19] and is characterized by emotional dependence and marked distress when the partner is perceived as unresponsive [5]. Attachment avoidance entails considering others as unavailable in times of need due to a negative model of others [19]. Attachment avoidance is characterized by reluctance to self-exposure, fear of intimacy, and excessive self-reliance [5]. Individuals high in attachment anxiety and/or avoidance are more insecurely attached.

The management of transgressions and interpersonal hurts through forgiveness has been widely studied from the attachment perspective [4,5,16]. Forgiveness can be conceptualized as a coping strategy in the face of an interpersonal hurt that implies a decrease in levels of resentment, and eventually an increase in feelings of benevolence toward the person who committed the offense [20,21]. More specifically, McCullough et al. [22] define forgiveness as a set of prosocial changes, where the offended person decreases their motivations to retaliate (take revenge) and takes distance from (avoidance) the person who has committed the offense and Increases their feelings of benevolence toward the offender.

Forgiveness can be understood in three ways. State or episodic forgiveness refers to the forgiveness of a particular offense in a specific interpersonal setting [22]. Trait or dispositional forgiveness refers to the relatively stable tendency to forgive in multiple circumstances and in a variety of relationships [23]. Finally, dyadic forgiveness is the inclination to forgive within a specific relationship [7], such as the couple relationship, which is the focus of this study.

Attachment theory provides a theoretical framework for understanding how people perceive and deal with transgressions and the related negative experiences [24]. The levels of security in attachment are linked to forgiveness, be it conceived as state, trait, or dyadic forgiveness [4,7]. For example, it has been reported that people who are more securely attached have a more constructive view of others and better emotional regulation strategies. They are more inclined to forgive a transgression, whereas people with higher levels of attachment anxiety and/or avoidance have greater difficulties in doing so [4,16,24,25].

Individuals with high attachment anxiety, due to their propensity to hyperactivate the attachment system, more easily experience relational hurts and tend to ruminate and make negative attributions in relation to a transgression [4,26,27,28], which would make them less inclined to forgive. Indeed, studies of individuals with higher attachment anxiety reveal that they are more prone to revenge, translated into actions aimed at damaging the partner as a way to “equal the score”, and tend to experience greater resentment and less benevolent feelings toward the offender [17,29].

On the other hand, people with high attachment avoidance, who tend to deactivate the attachment system and have a negative image of others, are more prone to show low empathy and negatively explain their partner’s behavior, using distancing as a self-protective strategy against relational transgressions [5,24]. Studies report that higher levels of attachment avoidance are associated with lower forgiveness [4,25], including greater revenge [16], a greater tendency to distance themselves from the couple when faced with a partner transgression [17], and lower benevolence and goodwill toward the offender [29].

However, the above-cited studies exclusively focused on heterosexual individuals. To our knowledge, no study has investigated the attachment–forgiveness link among LGB people. Only two studies have examined forgiveness in LGB individuals, showing that forgiveness is linked to variables such as shame and self-esteem [30], and psychobiological stress recovery [31], but neither study looked at associations with attachment.

Several lines of evidence suggest that attachment dynamics, core relational processes, and relational outcomes of same- and different-sex couples are fundamentally similar [32]. For example, higher levels of attachment anxiety and avoidance have been associated with lower relationship satisfaction and intimacy in the context of both heterosexual [17,33,34,35] and same-sex couples [8,36,37]. Higher attachment insecurities have been also linked to increased rates of heterosexual and same-sex intimate partner violence [36,38,39] and poorer abilities to manage conflicts effectively [8,40]. However, LGB couples experience unique challenges related to the marginalized status of their relationships [10], which can introduce barriers to the establishment of secure relationships, with detrimental effects on relational outcomes. For example, a recent study of same-sex couples reported that a minority stressor like internalized homonegativity (i.e., the internalization of negative attitudes toward one’s sexual orientation [10] can lead to lower relationship satisfaction via greater couple conflict [41]. Hence, although it appears that similarities in relationship functioning and dynamics outweigh differences across same- and different-sex couples, this still needs to be established for the attachment–forgiveness link. Investigating the relationship between attachment insecurity and forgiveness in individuals involved in same-sex couples could enrich our understanding of LGB couple relationships and their dynamics. This is relevant as it might assist clinicians in providing culturally competent care in clinical work with sexual minority individuals and couples [42].

### 1.2. The Role of Gender in Forgiveness

The role of gender in forgiveness has been extensively explored, but with inconsistent results. A meta-analysis of 70 studies revealed that women forgive more than men, at both state and trait levels [14]. This finding has also been observed in subsequent studies [43,44,45]. However, another meta-analytic review of 175 studies [15] did not find gender differences in episodic forgiveness, whereas other research reported higher levels of episodic [46] and dyadic forgiveness [47] in men. As for motivation toward revenge, several studies reported that the inclination to retaliate is greater in men than in women [28,29]. In addition, previous research concluded that women are more inclined toward benevolence after an interpersonal injury [14,48].

The evidence regarding gender differences in the link between attachment and forgiveness is scarce and referred exclusively to heterosexual individuals and couples. In Martin et al.’s dyadic study [16], it was observed that higher attachment avoidance predicted more revenge in reaction to transgressions in men only, but gender did not significantly moderate the effects of attachment insecurity on benevolence and avoidance motivations. In another study, no differences were detected in the association of attachment dimensions with avoidance and revenge motivations between male and female partners of heterosexual couples [17]. In a similar way, no gender differences were identified in the attachment–forgiveness link in a sample of divorced individuals [18].

Hence, gender appears to have a role in levels of forgiveness motivations. However, the question if the relationship between attachment and forgiveness is similar across gender requires further exploration. Altogether, gender differences in the associations between attachment insecurity and forgiveness have rarely been found, but there are still few studies, and no data are available regarding individuals involved in same-sex couples.

### 1.3. The Present Study

Considering all the above, the present study examines the association between attachment and forgiveness in LGB individuals involved in a same-sex relationship, and explores the moderating role of gender in this association. Different from previous research, which mostly focused on episodic forgiveness, this study will consider dyadic forgiveness, as it may reflect the dynamics of a couple’s relationship more closely than the handling of a specific offense.

We aim to ascertain whether same-sex couples do function similarly to different-sex couples, with specific reference to the relationship between attachment insecurity and dyadic forgiveness, in light of the minority stressors faced by LGB individuals and couples [10]. If indeed the similarities between same- and different-sex couples the outweigh differences [8,32], then we should expect that higher levels of attachment anxiety and avoidance are associated with a lower tendency to forgive within the couple relationship, as reported in studies of heterosexual individuals. More specifically, it should be expected that: higher levels of attachment anxiety are associated with a greater tendency toward revenge, higher levels of attachment avoidance are associated with a greater tendency toward avoidance and revenge, and lower levels of both attachment anxiety and avoidance are associated with a greater inclination toward benevolence. Based on the paucity of studies that examined gender-specific associations between attachment and forgiveness, and given inconsistent evidence of the role of gender in forgiveness levels, no specific hypothesis was proposed on this point, which was examined in a purely exploratory manner.

To achieve the study aims, structural equation modeling (SEM) was used, including attachment anxiety and attachment avoidance as the independent variables and dyadic forgiveness (avoidance, revenge, and benevolence motivations) as the outcome variables. Invariance of the associations between attachment insecurity and dyadic forgiveness across gender was tested using multigroup SEM.

## 2. Materials and Methods

### 2.1. Design and Procedure

This correlational study is part of a larger research project on same-sex couple functioning. Inclusion criteria were being 18 years of age or older, self-defining as gay, lesbian, or bisexual, and having been in a same-sex relationship for at least 6 months. Recruitment was carried out using non-probabilistic sampling by quotas according to age and sex. Participants were asked to complete an online questionnaire, administered through the Survey Monkey platform. The initial page of the online questionnaire contained a detailed description of the research objectives, conditions for participation, and information on the confidentiality and privacy of the data. To start the survey, the participants had to click on an informed consent button reading, “Yes, I agree to participate”. The participants received financial compensation for the time spent answering the questionnaire. The present study was approved by the Ethics Committee of the Universidad Católica del Norte, following the ethical standards for working with human beings.

### 2.2. Participants

A total of 248 individuals met the inclusion criteria and agreed to participate. The participants were recruited from the northern (23%), central (46.8%), and southern areas of Chile (30.2%). Participants were 131 women (52.8%) and 117 men (47.2%), aged between 18 and 67 years (M = 31.02, SD = 9.39). About 60% of participants (*n* = 148) had higher (technical or university) education, and 62.9% (*n* = 156) were employed. Forty-two percent (*n* = 105) self-identified as gay, 40.7% (*n* = 101) as lesbian, and 16.9% (*n* = 42) as bisexual. Seven percent of participants (*n* = 18) reported having children. Relationship length ranged between 6 months and 36 years (M = 4.17 years, SD = 5.04), and 57.7% of participants (*n* = 143) were cohabiting with their partner.

### 2.3. Measures

The online questionnaire included a first section collecting information on sex, age, educational level, job status, sexual orientation, presence of children, relationship length, and cohabitation status. Respondents indicated their sexual orientation by answering a multiple-choice question with response options gay, lesbian, bisexual, and other.

Attachment insecurity was measured using the 12-item Spanish validated version [49] of the Experiences in Close Relationships Questionnaire (ECR) [50], a widely used self-report measure of adult attachment. The ECR has two 6-item subscales of attachment anxiety (e.g., “I worry that my partner is not as interested in me as I am in her”) and attachment avoidance (e.g., “I feel uncomfortable opening up to my partner”). Items are rated on a 7-point scale (1 = totally disagree to 7 = totally agree), with higher scores indicating higher levels of attachment insecurity. The short version of the ECR used in this study was originally validated using five samples, including individuals involved in same-sex relationships and heterosexual couples. Evidence of validity and reliability support its use with both same- and different-sex couples [51]. The Spanish ECR showed good psychometric properties, with expected associations with measures of difficulties in emotion regulation, empathy, psychological distress, and life satisfaction, and Cronbach’s αs from 0.72 to 0.83 for the attachment anxiety subscale and 0.78 to 0.89 for the attachment avoidance subscale [49]. Reliability in the present study was α = 0.81 for attachment anxiety and α = 0.76 for attachment avoidance.

Dyadic forgiveness was assessed using a 14-item Chilean validated version [46] of the Transgression-Related Interpersonal Motivations Inventory (TRIM) [52]. The original TRIM assesses a person’s reaction to a specific transgression (i.e., episodic forgiveness). For the purposes of the present study, the TRIM instructions were modified so that the participants were asked to answer items thinking about situations in which they felt hurt or treated unfairly by their current partner, and the thoughts and feelings usually experienced in such situations. The Chilean TRIM used in this study is made up of three subscales: motivation to avoid the person who committed the offense (6 items; e.g., “I avoid him/her”), motivation to seek revenge (4 items; e.g., “I want to see him/her hurt and miserable”, and benevolence motivation (4 items; e.g., “I want us to bury the hatchet and move forward with our relationship”). Items are rated on a 5-point scale (1 = totally disagree to 5 = totally agree), with a higher score reflecting higher levels of forgiveness motivations. The Chilean TRIM showed adequate internal consistency, with a Cronbach’s α higher than 0.70 for each subscale, and appropriate validity as evidenced by associations in the expected directions with global self-report of forgiveness, relationship satisfaction, and socioemotional adjustment [46]. In this study, Cronbach’s α was 0.80 for avoidance, 0.65 for revenge, and 0.79 for benevolence.

### 2.4. Data Analysis

Analyses were performed in three steps. First, preliminary analyses were conducted, including Pearson’s correlations between the study variables, ANOVA to compare scores in the study variables between women and men, and tests of potential covariates. To select covariates, the outcome variables were correlated with age and relationship length and compared across groups based on education (i.e., up to secondary or higher education), job status (i.e., employed or unemployed), sexual orientation (i.e., gay, lesbian, or bisexual), and cohabitation status (i.e., cohabiting or not) using ANOVA. Only variables significantly and at least moderately associated (*r* ≥ |0.30| or *d* ≥ 0.50), with the outcome variables were included as covariates [53].

Second, confirmatory factor analysis (CFA) was used to test the two- and three-factor measurement models of ECR and TRIM, respectively, on the total sample. We then applied multigroup CFA to test the measurement invariance across gender of attachment insecurity and forgiveness measures. Indeed, metric invariance (i.e., equal factor loadings) needs to be established prior to testing for invariance of structural path coefficients across groups [54]. To test for measurement invariance, configural (equal factor structure) and metric (equal factor loadings) invariance models were compared. Metric invariance was considered achieved if constraining the factor loadings as equal across gender produced a nonsignificant decrease in model fit, as indicated by a nonsignificant *χ*^2^ difference test (Δ*χ*^2^), and sufficiently small changes in model fit indices, as indicated by a decrement in the comparative fit index (ΔCFI) of ≤ 0.010, and increases in root mean square error of approximation (ΔRMSEA) and standardized residual mean root (ΔSRMR) of ≤ 0.015 and ≤ 0.010, respectively [55]. If full metric invariance was not achieved, partial metric invariance was considered, by sequentially relaxing equality constraints on factor loadings to identify noninvariant items [56]. Nested models were compared by means of Δ*χ*^2^ tests. The effects-coding identification method was used in measurement invariance testing [57].

In the third step of the analyses, we tested the proposed model of attachment insecurity predicting dyadic forgiveness using SEM. Parcels were used as latent variable indicators to reduce the number of estimated parameters [58]. To test the invariance of structural paths across gender, multigroup modeling was applied. We first estimated a two-group fully unconstrained model in which all structural parameters were allowed to vary across gender, then estimated a constrained model in which these parameters were all set to be equal across gender. Factor loadings were constrained to equality across gender. A Δ*χ*^2^ test was used to compare the fit of the fully unconstrained and constrained models. A nonsignificant Δ*χ*^2^ test would indicate no moderating effect of gender. In the case of a significant Δ*χ*^2^ test, a series of follow-up tests were conducted on individual constraints to determine which structural paths were gender noninvariant.

In both CFA and SEM analyses, model fit was evaluated based on RMSEA and SRMR, with values of ≤0.08 indicating acceptable fit and values of ≤0.05 indicating close fit, and CFI, with values ≥ 0.90 and ≥0.95 as indicative of acceptable and close fit, respectively [59]. A sample size of at least 200 participants was established a priori following recommendations for reasonable sample sizes in social and behavioral sciences research using SEM techniques [60]. In addition to statistical significance (*p* < 0.05) we considered effect size, with Pearson’s *r* and standardized path coefficients of 0.10 interpreted as small, 0.30 medium, and 0.50 large, and Cohen’s *d* of 0.20 interpreted as small, 0.50 medium, and 0.80 large [61]. CFA and SEM analyses were conducted using maximum likelihood in Mplus 8.4. All other analyses were performed using IBM SPSS 27.

## 3. Results

### 3.1. Preliminary Analyses

As shown in Table 1, attachment anxiety was significantly, positively correlated with avoidance and revenge motivations, with small-to-medium and small effect sizes, respectively, and unrelated to benevolence motivation. Attachment avoidance correlated significantly and positively with avoidance and revenge motivations, and negatively with benevolence motivation, with medium effect sizes. Attachment anxiety and avoidance were weakly-to-moderately positively correlated. Intercorrelations between dyadic forgiveness motivations were of medium effect size.

#### 3.1.1. Gender Differences in the Study Variables

Gender comparisons indicated that, compared to women, men showed slightly higher attachment anxiety (*F*(1,246) = 7.26, *p* = 0.008, *d* = 0.34) and moderately higher attachment avoidance (*F*(1,246) = 20.90, *p* < 0.001, *d* = 0.59) and revenge motivation (*F*(1,246) = 27.16, *p* < 0.001, *d* = 0.59). There were no gender differences in avoidance (*F*(1,246) = 3.34, *p* = 0.07, *d* = 0.23) and benevolence motivations (*F*(1,246) = 3.66, *p* = 0.06, *d* = 0.24). Descriptive statistics of the study variables by gender are displayed in Table 2.

#### 3.1.2. Testing of Potential Covariates

Age was unrelated to dyadic forgiveness, whereas relationship length was significantly, positively correlated with benevolence motivation, with a small effect size (Table 1). Results of ANOVAs (Table S1) indicated that mean scores in forgiveness motivations did not vary depending on education, job status, or cohabitation status. There was a significant effect of sexual orientation on revenge motivation. Post-hoc pairwise comparisons (Bonferroni) indicated that gay participants reported significantly, moderately higher revenge motivation than lesbian participants (*p* < 0.001, *d* = 0.56). Sexual orientation was thus incorporated as a covariate in the SEM.

#### 3.1.3. Metric Invariance across Gender of Attachment Insecurity and Dyadic Forgiveness Measures

The goodness of fit of the ECR two-factor model was below acceptable levels (*χ*^2^(53) = 156.67, *p* < 0.001, RMSEA = 0.09, SRMR = 0.06, CFI = 0.89). Inspection of modification indices indicated that allowing the uniqueness of two items covary (i.e., Item 2—“I worry about being abandoned” with Item 3—“I worry that romantic partners won’t care about me as much as I care about them”) would improve model fit. The model was thus respecified including this covariation to account for similar item wording. The goodness of fit of the respecified model improved significantly (Δ*χ*^2^(1) = 57.95, *p* < 0.001), with goodness-of-fit indices indicating close fit (*χ*^2^(52) = 98.71, *p* < 0.001, RMSEA = 0.06, SRMR = 0.05, CFI = 0.95). This model was tested for invariance across gender. As shown in Table 3, multigroup CFAs indicated that full metric invariance was not supported for the ECR. Subsequent analyses revealed that the factor loadings of Item 5 (“I am nervous when partners want to get too close to me”) were noninvariant (Δ*χ*^2^(1) = 10.90, *p* = 0.001). The equality constraint on the loading of this item was thus released, which led to achievement of partial metric invariance.

The TRIM three-factor model showed an acceptable fit to the data (*χ*^2^(74) = 161.90, *p* < 0.001, RMSEA = 0.07, SRMR = 0.06, CFI = 0.92). Testing for invariance across gender via multigroup CFA indicated that full metric invariance was not achieved for the TRIM. Subsequent analyses showed that the factor loadings of Item 9 (“I prefer not to be too close to my partner”) differed across gender (Δ*χ*^2^(1) = 13.75, *p* < 0.001). When the equality constraint on this factor loading was released, partial metric invariance was supported (Table 3). Achievement of partial metric invariance for both the attachment insecurity and dyadic forgiveness measures allowed for a meaningful comparison of structural path coefficients across gender in subsequent SEM analyses.

### 3.2. Testing of Hypothesized Associations with SEM

In the total sample, the hypothesized model of attachment insecurity dimensions predicting dyadic forgiveness motivations showed a close fit to the data (*χ*^2^(79) = 118.64, *p* = 0.003, RMSEA = 0.05, SRMR = 0.06, CFI = 0.97). Higher attachment anxiety was associated, with a small effect size, with a higher tendency to avoid the partner (*β* = 0.16, *SE* = 0.08, *z* = 2.12, *p* = 0.03), whereas it was nonsignificantly associated with revenge and benevolence motivations (*β* = 0.10, *SE* = 0.08, *z* = 1.12, *p* = 0.25 and *β* = 0.09, *SE* = 0.08, *z* = 1.13, *p* = 0.26). Higher attachment avoidance was linked, with moderate effect sizes, with higher avoidance (*β* = 0.36, *SE* = 0.08, *z* = 4.87, *p* < 0.001) and revenge motivations (*β* = 0.33, *SE* = 0.09, *z* = 3.53, *p* < 0.001) and with a lower degree of benevolence toward the partner (*β* = -0.415, *SE* = 0.08, *z* = -5.20, *p* < 0.001) (Figure S1).

**Figure 1:**
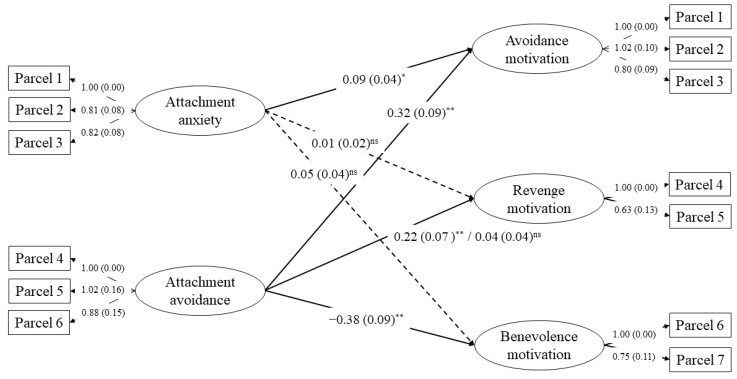
Final model with the noninvariant pathway freely estimated across gender. Note. Unstandardized estimates (standard errors) are shown. Correlations between variables, measurement errors and residuals are not shown to simplify presentation. Solid lines represent significant paths; dashed lines represent nonsignificant paths. Estimates before and after the slash refer to men and women, respectively. All factor loadings are significant at *p* < 0.001. ^ns^ *p* > 0.05. * *p* < 0.05. ** *p* ≤ 0.001.

Multigroup SEM was used to test for invariance of structural paths across gender. The Δ*χ*^2^ test comparing the two-group fully unconstrained and constrained models was significant (Δ*χ*^2^(6) = 15.95, *p* = 0.01), suggesting a moderating effect of gender. Follow-up tests revealed that the association between attachment avoidance and revenge motivation noninvariant across women and men (Table 4). Among men, higher attachment avoidance was significantly associated with higher revenge motivation (*z* = 2.81, *p* = 0.008), while this association was nonsignificant among women (*z* = 0.87, *p* = 0.38). All other structural paths were gender invariant, indicating that the associations found in the overall sample between attachment insecurity dimensions and avoidance and benevolence motivations, and between attachment anxiety and revenge motivation generalized across women and men. The fit of the final model (Figure 1), with the pathway from attachment avoidance to revenge motivation freely estimated across gender, was excellent (*χ*^2^(126) = 141.76, *p* = 0.16, RMSEA = 0.03, SRMR = 0.06, CFI = 0.99).

## 4. Discussion

This study aimed to clarify whether the notion that same-sex couples function similarly to different-sex couples also applies to the relationships between attachment insecurities and dyadic forgiveness, which were examined in a sample of LGB individuals involved in a same-sex relationship.

Results of SEM analyses indicated that attachment anxiety was positively associated with the tendency to avoid and distance oneself from the partner after a transgression and was unrelated to motivations toward revenge and benevolence, with no differences in these associations across gender. Attachment avoidance was associated positively with avoidance motivation and negatively with benevolence motivation, regardless of gender. However, higher attachment avoidance was linked to higher revenge motivation among men only.

The finding that attachment anxiety was associated with avoidance motivation differs from previous studies conducted with heterosexual couples, where attachment anxiety was not related to avoidance motivation [16,17]. A possible explanation for such a difference between heterosexual and LGB individuals is exposure to stigma-related stress in the latter. More anxiously attached individuals in our LGB sample showed a higher tendency to avoid physical and emotional contact with the partner probably due to their tendency to experience intrusive rumination (i.e., an excessive focus on negative thoughts and feelings about past transgressions) [26] and make negative attributions (i.e., more negative interpretations of hurtful behaviors) [4,27] in response to transgressions. Indeed, there is evidence that exposure to sexual minority stressors enhances emotional dysregulation, including engagement in rumination, and cognitive processes such as pessimistic attributions [62,63,64].

In addition, different from findings in heterosexual couples [17], non-significant associations were detected in our LGB sample between attachment anxiety and revenge and benevolence motivations. It is likely that a heightened ambivalence between anger and fear of abandonment, which are characteristic of anxiously attached individuals, nullifies the effects of attachment anxiety toward revenge and benevolence among LGB individuals. Indeed, it has been reported that sexual minority groups may experience increased sensitivity to anger coupled with an intense fear of rejection as a consequence of chronic experiences of marginalization [10,62,65].

As for attachment avoidance, we found that, regardless of gender, higher levels of attachment avoidance were associated with a greater tendency to avoid the partner after a transgression. This finding is consistent with previous evidence in heterosexual individuals [5,17,24]. Given that people with high attachment avoidance have a negative view of others and make fewer positive interpretations about the other’s behaviors [4], it is expected that when their partners commit a transgression, they will use avoidance, as this guarantees them self-protection and low self-exposure [17]. Consequently, those who are more prone to attachment avoidance, prioritize self-confidence and emotional distancing after transgressions instead of involving in interactions oriented to restoring harmony and closeness [29].

We found that, for both women and men, higher attachment avoidance was associated with less goodwill and more positive feelings toward one’s partner after transgressions, consistent with previous studies of heterosexual individuals. This finding is coherent with evidence that attachment avoidance is predictive of less empathy toward the partner [24,66], due to the deactivation of the attachment system and the negative model of others [19], which may lead to lower benevolence toward the partner after a transgression. In addition, as benevolence reflects a security-based attachment strategy based on a positive view of others, it is expected that more avoidantly attached individuals will demonstrate lower levels of benevolent feelings [16].

An association between attachment avoidance and revenge motivation was found among men but not among women, similar to a previous study of heterosexual couples [16], but different from another study of heterosexual couples, where no significant association was detected between attachment avoidance and revenge [17]. On the one hand, it is likely that men with high attachment avoidance, who hold a negative view of others, express more retaliation desires and hostile attitudes toward the partner in the face of transgressions because these characteristics are more accepted according to gender roles and are congruent with the different facets of attachment avoidance. This is in line with Miller et al.’s [14] meta-analytic study, where larger gender differences were detected in vengeance than in any other forgiveness–related measure. On the other hand, it is possible that avoidantly attached women suppress their desires for revenge toward the partner [5,27], prioritizing strategies other than seeking revenge, an aspect that should be explored in future studies. In addition, the motivation for revenge implies harboring hostile attitudes toward the partner, which is more characteristic of men [67], and is coupled with a high degree of attachment avoidance when facing negative relational events [29].

As for gender differences in the mean levels of the study variables, we found that men reported higher scores than women in both attachment anxiety and attachment avoidance. The gender difference in attachment avoidance is consistent with previous studies of both heterosexual and sexual minority populations [68], and might be attributable to the internalization of traditional male gender roles associated with less emotional involvement [69,70]. The difference in attachment anxiety is consistent with a study of same-sex couples [8], yet differs from evidence in the heterosexual population, where attachment anxiety is usually higher in women [5]. The higher levels of attachment anxiety observed in men could be understood from the social belief that people involved in same-sex couples transgress traditional gender roles [71]. In addition, there is a greater probability that gay and bisexual men have suffered from homophobic acts than lesbian women [72], a finding also replicated in the Chilean context, where gay men are targets of heterosexist attitudes more than lesbian women [73]. Experiences of discrimination may contribute to the development of negative self-schemas, including negative representations of self and others, potentially increasing the risk of experiencing higher levels of attachment insecurity [62].

Men in our study also reported higher revenge motivation than women, in line with numerous studies that have associated the search for revenge with the male gender [14,16,43,44,74]. This could be related to the greater social legitimation of this motivation according to traditional gender roles, whereas women may feel more restricted to openly expressing the motivation for revenge, being able to use subtler forms of vindictive behavior [44]. However, most studies were conducted with heterosexual individuals. No gender differences were observed in avoidance and benevolence motivations, which is coherent with previous studies of heterosexual individuals [16,43,44]. Given these results, future studies should consider investigating a possible moderating role of sexual orientation in the link between gender and motivation toward revenge.

Altogether, for the associations between attachment avoidance and dyadic forgiveness, we found that the similarities between individuals in same- and different-sex relationships outweighed the differences. For attachment anxiety, our results indicated peculiarities of associations with dyadic forgiveness in LGB relative to heterosexual individuals. We have offered interpretations for these differences, which nonetheless need to be confirmed by further studies. From a cultural point of view, despite that in today’s Chile there is greater acceptance of sexual diversity and more recognition of LGB rights (i.e., a law allowing same-sex marriage was recently approved), same-sex couples maintain their relationships in a cultural context that favors heterosexual relationships. Indeed, LGB people in Chile are still victims of violence and discrimination [75], which are related to negative mental health and relational outcomes in this group [76,77]. Hence, the findings of the present study are especially relevant in light of the particularities of the Chilean context.

The present research has certain limitations that offer, at the same time, directions for future research. First, the correlational design of this study precludes any conclusion about causal relationships. Longitudinal replication studies are therefore encouraged. Second, the sample was mostly made up of highly educated young people, which limits the generalizability of the results. Future research should therefore include larger samples covering different age groups and educational levels. Third, we did not ask for the number, severity, or type (e.g., criticism, infidelity) of the transgressions recalled, which have a role in unforgiveness [16,78]. Fourth, the number of bisexual participants was small compared to gay and bisexual participants. This did not allow us to test for invariance across sexual orientations, which should be considered in future research. Fifth, this study exclusively focused on individuals with a same-sex partner. Considering that some of our results differed from those reported in previous studies of heterosexual individuals, future research should more specifically make comparisons between same- and different-sex couples. In addition, we did not measure variables that may influence the relationships in our model. Indeed, LGB individuals are subject to specific stressors because of their sexual orientation [79]. Therefore, it would be important that future studies also consider variables such as internalized homonegativity or experiences of discrimination faced as a couple. Finally, this was an individual-based study. Couple-based studies of LGB samples are warranted to examine the dyadic effects of attachment orientations on conflict management strategies such as dyadic forgiveness.

The findings of this study also have clinical implications. Understanding how attachment facilitates or impedes forgiveness after a transgression would provide couple therapists with the knowledge to promote the resolution of transgressions in the context of couple relationships. Fostering the development of more secure attachment bonds in a clinical context may be relevant for the management of transgressions. This would be especially relevant for clients involved in a same-sex relationship, as LGB individuals experience higher levels of stress associated with their minority status [10].

## 5. Conclusions

This is the first study to adopt the attachment theory perspective to investigate dyadic forgiveness in the context of same-sex couples, revealing that attachment avoidance plays a more prominent role in the tendency to forgive in same-sex couples. Research on factors that contribute to understanding the dynamics of couple relationships in LGB people is scarce in Latin America. Therefore, this study opens a line of research to gain a better understanding of couple relationships in sexually diverse populations.

## Figures and Tables

**Table 1:** Correlations for study variables and potential covariates.

Variable	1	2	3	4	5
1. Attachment anxiety	-				
2. Attachment avoidance	0.25 ***	-			
3. Avoidance motivation	0.25 ***	0.34 ***	-		
4. Revenge motivation	0.18 **	0.30 ***	0.41 ***	-	
5. Benevolence motivation	−0.04	−0.30 ***	0.41 ***	−0.34 ***	-
6. Age	−0.12	0.01	−0.12	−0.03	0.10
7. Relationship length	−0.15 *	−0.19 **	−0.11	−0.09	0.16 *

* *p* < 0.05. ** *p* < 0.01. *** *p* < 0.001.

**Table 2:** Descriptive statistics of the study variables.

Variable	Women (*n* = 131)	Men (*n* = 117)	Total (*n* = 248)
Attachment anxiety	20.08 (7.94)	22.91 (8.60)	21.42 (8.36)
Attachment avoidance	10.31 (5.10)	13.62 (6.26)	11.87 (5.90)
Avoidance motivation	12.09 (4.95)	13.28 (5.31)	12.65 (5.15)
Revenge motivation	4.49 (1.08)	5.56 (2.07)	5.00 (1.71)
Benevolence motivation	16.66 (3.09)	15.90 (3.21)	16.30 (3.17)

Note. Mean (standard deviation). Total score range was 6 to 42 for attachment anxiety and attachment avoidance, 6 to 30 for avoidance motivation, and 4 to 20 for revenge and benevolence motivations.

**Table 3:** Measurement invariance testing of ECR and TRIM.

Level of Invariance	*df*	*χ* ^2^	Δ*df*	Δ*χ*^2^	CFI	ΔCFI	RMSEA	ΔRMSEA	SRMR	ΔSRMR
ECR										
Configural	104	159.21	-	-	0.941	-	0.065	-	0.059	-
Metric	114	182.76	10	23.54 **	0.927	0.014	0.070	0.005	0.073	0.014
Metric partial ^a^	113	171.86	9	12.65 ^ns^	0.937	0.004	0.065	0.000	0.069	0.010
TRIM										
Configural	148	240.72	-	-	0.911	-	0.071	-	0.071	-
Metric	159	264.16	11	23.45 *	0.899	0.012	0.073	0.002	0.082	0.011
Metric partial ^b^	158	250.42	10	9.70 ^ns^	0.912	0.001	0.069	0.002	0.078	0.007

^ns^*p* > 0.05. * *p* < 0.05. ** *p* < 0.01. ^a^ Loading of Item 5 freely estimated across gender. ^b^ Loading of Item 9 freely estimated across gender.

**Table 4:** Multigroup analyses on gender invariance of structural paths.

Path	Women (*n* = 131)	Men (*n* = 117)	Δ*χ*^2^(1)	*p*
Attachment anxiety → Avoidance motivation	0.10 (0.06)	0.09 (0.06)	0.011	0.916
Attachment avoidance → Avoidance motivation	0.22 (0.12)	0.41 (0.12)	1.544	0.214
Attachment anxiety → Revenge motivation	0.01 (0.02)	0.03 (0.04)	0.101	0.751
Attachment avoidance → Revenge motivation	0.03 (0.04)	0.22 (0.08)	5.052	0.025
Attachment anxiety → Benevolence motivation	−0.02 (0.07)	0.10 (0.06)	1.468	0.226
Attachment avoidance → Benevolence motivation	−0.54 (0.20)	−0.30 (0.12)	1.500	0.221

Note. Unstandardized path estimates (standard errors) from the two-group fully unconstrained model are reported.

## Data Availability

Although the data are not publicly available due to privacy and ethical restrictions, they are available, upon reasonable request, from the corresponding author.

## Supplementary Materials

The following supporting information can be downloaded at: https://www.mdpi.com/article/10.3390/ijerph191811152/s1, Table S1: Dyadic forgiveness mean scores (SD) by groups based on potential covariates; Figure S1: SEM testing of the hypothesized associations in the total sample.
