# Supplementary material for: Attachment and Dyadic Forgiveness in Individuals in Same-Sex Couple Relationships
Source: Int J Environ Res Public Health. 2022 Sep 6;19(18):11152. doi: 10.3390/ijerph191811152 (PMC9517675; doi:10.3390/ijerph191811152)
Supplement: Supplementary file 1 [file ijerph-19-11152-s001.zip › ijerph-1887235-supplementary.pdf]

Table S1. Dyadic forgiveness mean scores (SD) by groups based on potential covariates.

**Variable**	***n* (%)**	**Avoidance motivation**	**Revenge motivation**	**Benevolence motivation**
Education				
Up to secondary	100 (40.3)	12.89 (5.39)	4.97 (1.60)	16.22 (3.26)
Higher	148 (59.7)	12.49 (4.99)	5.01 (1.78)	16.36 (3.11)
$F(1,246)$		0.35, $p = 0.55$	0.04, $p = 0.84$	0.11, $p = 0.74$
Job status				
Employed	156 (62.9)	12.34 (5.08)	4.92 (1.64)	16.50 (3.17)
Unemployed	92 (37.1)	13.18 (5.23)	5.12 (1.81)	15.97 (3.14)
$F(1,246)$		1.56, $p = 0.21$	0.77, $p = 0.38$	1.64, $p = 0.20$
Sexual orientation				
Gay	105 (42.3)	13.25 (5.46)	5.50 (2.05)	15.91 (3.28)
Lesbian	101 (40.7)	11.65 (4.73)	4.49 (1.14)	16.77 (3.16)
Bisexual	42 (16.9)	13.57 (5.02)	4.95 (1.55)	16.14 (2.81)
$F(1,173)$		3.34, $p = 0.04$	9.87, $p < 0.001$	1.97, $p = 0.14$
Cohabitation status				
Cohabiting	143 (57.7)	12.46 (5.26)	4.94 (1.66	16.44 (3.21)
Not cohabiting	105 (42.3)	12.91 (5.00)	5.08 (1.77)	16.11 (3.11)
$F(1,246)$		0.47, $p = 0.50$	0.40, $p = 0.53$	0.64, $p = 0.42$

Note. SD = standard deviation. Total score range was 6 to 42 for attachment anxiety and attachment avoidance, 6 to 30 for avoidance motivation, and 4 to 20 for revenge and benevolence motivations.

Figure S1. SEM testing of the hypothesized associations in the total sample.

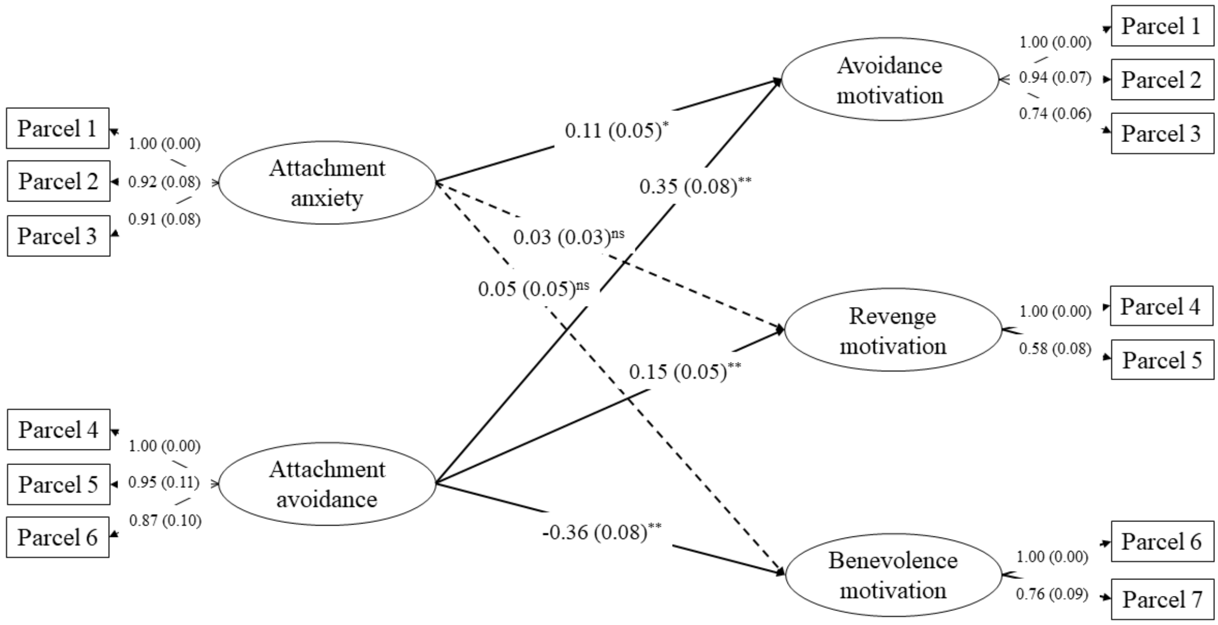

Note. Unstandardized estimates (standard errors) are shown. Correlations between variables, measurement errors and residuals are not shown to simplify presentation. Solid lines represent significant paths; dashed lines represent nonsignificant paths. All factor loadings are significant at $p < 0.001$.

^{ns} $p > 0.05$.

* $p < 0.05$.

** $p \leq 0.001$.
